# Epidemiology of Brain Abscess: A Retrospective Cohort From a Neurosurgical Tertiary Referral Center in England

**DOI:** 10.1093/ofid/ofaf655

**Published:** 2025-10-21

**Authors:** Victoria B Allen, Ahmed Raslan, Ann Maria Muuli, Noor Yehya Alkhafaji, Katie Bechman, Kankanange Don Dulnie Saranga Wijeweera, Ali Abdulla, Mohammad Baraka, Vindhya Prasad, Anjaneya Bapat, Keyoumars Ashkan

**Affiliations:** Centre for Rheumatic Diseases, King's College London, London, UK; Department of Neurosurgery, King's College Hospital, London, UK; GKT School of Medical Education, Faculty of Life Sciences & Medicine, King's College London, London, UK; GKT School of Medical Education, Faculty of Life Sciences & Medicine, King's College London, London, UK; Centre for Rheumatic Diseases, King's College London, London, UK; Department of Microbiology, Faculty of Medicine, University of Ruhuna, Matara, Sri Lanka; GKT School of Medical Education, Faculty of Life Sciences & Medicine, King's College London, London, UK; Neurosurgical Department, KasrAlainy Faculty of Medicine, Cairo University, Cairo, Egypt; Department of Neurosurgery, King's College Hospital, London, UK; Department of Microbiology & Infectious Diseases, King's College Hospital, London, UK; Department of Neurosurgery, King's College Hospital, London, UK; School of Neuroscience, Institute of Psychology, Psychiatry and Neuroscience, King's College London, London, UK; School of Biomedical Engineering and Imaging Sciences, King's College London, London, UK

**Keywords:** bacteriology, brain abscess, central nervous system infections, neurosurgery, suppuration

## Abstract

**Background:**

Brain abscess is a serious infection with substantial morbidity and mortality. Current data on the etiology, management, and outcomes are limited. This study describes the epidemiology of brain abscess in a large patient cohort.

**Methods:**

This is a retrospective, observational study of brain abscess at a tertiary referral center. Patients were selected using hospital coding. Demographic, clinical, neurosurgical, and microbiological data were analyzed.

**Results:**

We identified 174 patients with brain abscess admitted between 2012 and 2023 (32 pediatric, 142 adults, 66.7% male). Subdural empyema and parenchymal abscess were the most common abscess types in the pediatric and adult cohorts, respectively. A microbiological diagnosis was made in 74.1% of cases. *Staphylococcus aureus* was the most common cause of postsurgical brain abscess, causing 27.8% of these cases. *Streptococcus anginosus* caused 42.8% of community-acquired brain abscesses. Microbiological samples were sent for 16S rRNA gene polymerase chain reaction (PCR) testing in 33 cases (19.0%). A new microbiological diagnosis was made in 14 of these 33 cases (42.4%). In-hospital mortality was 13.4%. Increasing age and poor admission Glasgow Coma Score were significantly associated with mortality. A trend toward decreasing mortality was seen with *S. anginosus*.

**Conclusions:**

Our data reinforce the importance of early diagnosis and multidisciplinary management, particularly in older patients. Molecular diagnostics, including 16S rRNA gene PCR, may play an increasing role in guiding treatment in the future.

Brain abscess is a serious intracerebral infection that begins as a localized area of cerebritis and develops into a collection of pus surrounded by a capsule. There were an estimated 2.86 first-time admissions per 100 000 population with brain abscess in England in 2019, and the incidence has been rising over the past 2 decades [1].

Before the widespread adoption of modern antibiotics, diagnostic imaging, and neurosurgical techniques, brain abscess was almost universally fatal. Reported mortality rates in modern patient cohorts are around 15%–20% [2, 3] but are notably lower in pediatric populations [4]. Mortality rates may be as high as 50% in vulnerable groups, such as following solid organ transplantation [5]. Around 70% of patients make a full recovery, with the remaining 30% experiencing varying degrees of neurological sequelae [3]. Epilepsy occurs in around 30% of patients following a brain abscess [6].

Brain abscesses may occur by direct spread from a contiguous focus of infection or via hematogenous spread from a distant infectious focus. They may also occur as a postoperative complication of neurosurgery or following intracranial trauma [7]. Important risk factors for brain abscess include ear, nose, and throat infections, dental infections, malignancy, neurosurgery, and immunomodulatory therapies [8]. Brain abscesses may be caused by a wide range of pathogens including bacteria, mycobacteria, fungi, helminths, and protozoa. Staphylococci and streptococci have been identified as the most common causative pathogens [7], but these vary according to the underlying etiology of infection.

Neurosurgical drainage and high-dose antibiotics represent the cornerstones of brain abscess management. Despite these, a paucity of randomized clinical trials and historical lack of clinical guidelines have led to considerable variation in clinical practice [9]. A recent guideline published by the European Society of Clinical Microbiology and Infectious Diseases (ESCMID) aimed to address this gap [10]. Surgical management includes aspiration or excision of the abscess. The choice of surgical procedure has been a matter of debate and depends on the patient's clinical status, the neuroradiographic characteristics of the abscess, and the experience and expertise of the treating surgeon. Aspiration may be more appropriate for abscesses in the cerebritis stage, deep-seated abscesses, or abscesses in eloquent areas of the brain where excision would be inappropriate. Excision may be required in infections caused by resistant pathogens, for multiloculated abscesses, or where aspiration has failed [11].

Previously published studies of patients with brain abscess in England have been small, with most including <100 patients and 1 study including 113 patients [4, 12–15]. This study aimed to describe the epidemiology of brain abscess in a large cohort of patients admitted to a teaching hospital and tertiary referral center for neurosurgery in England over a 10-year period.

## METHODS

### Data Collection and Extraction

Data were collected retrospectively from electronic hospital records of patients treated at our center. Patients were identified using International Classification of Diseases, 10th Revision (ICD-10), codes G.06 and G.06.2 pertaining to “intracranial abscess and granuloma” and “extradural and subdural abscess, unspecified,” respectively. We did not include ICD-10 codes B58.2 or B58.9, which pertain to cerebral toxoplasmosis, in our study. The search was performed with assistance from the Trust Business Intelligence Unit. All patients admitted between 01/01/2012 and 12/31/2023 were included. Patient records underwent initial screening to remove any erroneously coded cases of brain abscess. There were no age restrictions. Included cases were reviewed, and relevant data were extracted using a predesigned proforma.

### Clinical Data

Data were collected on patient demographics including age and sex and Glasgow Coma Score (GCS) on admission. Data were also collected on predisposing factors including the presence or absence of dental or sinus infection, diabetes mellitus, intravenous drug use, alcohol dependence, human immunodeficiency virus (HIV) infection, and malignancy. Data on the type of diagnostic imaging performed were collected, specifically whether computed tomography (CT) scanning, magnetic resonance imaging (MRI), or both were performed. Data on the type of brain abscess diagnosed on imaging were collected. Neurosurgical data included the type of surgery performed. Outcome data included Glasgow Outcome Score (GOS) and in-hospital mortality.

### Microbiological Data

Microbiological samples were processed in the microbiology laboratory in accordance with UK Standards for Microbiology Investigations (UK SMIs). Relevant samples included abscess pus or tissue, intraoperative swabs, and blood cultures.

Pus samples from brain abscess cases were handled according to the following protocol: An initial gram stain was performed on arrival in the laboratory. The sample was then inoculated onto (1) cooked meat enrichment broth with extended incubation for 10 days under aerobic conditions, (2) blood and chocolate agar in 5% CO^2^ and MacConkey agar in air, (3) blood, and neomycin fastidious anaerobe agar under aerobic conditions with metronidazole discs. Plates were incubated at 35°C–37°C. Any growth from these plates was analyzed by matrix-assisted laser desorption/ionization–time of flight (MALDI-TOF) mass spectrometry. Specific laboratory testing for additional rarer pathogens was undertaken according to the instructions of the clinical microbiologist involved in the case.

Antibiotic susceptibility testing was undertaken utilizing a combination of antibiotic disc susceptibility testing, minimum inhibitory concentration (MIC) test strips, and the Biomerieux Vitek 2 automated system. Clinical breakpoints were interpreted as per the most recent European Committee on Antimicrobial Susceptibility Testing (EUCAST) guidelines at the time of testing. Data on antibiotic treatment were not available. In our institution, empirical treatment of community-acquired brain abscess consists of ceftriaxone and metronidazole, and postsurgical cases are treated with meropenem and vancomycin. These regimens are then narrowed or changed if appropriate based on culture results. These antibiotic regimens remained consistent over the study period.

16S rRNA gene polymerase chain reaction (PCR) and other forms of send-away PCR testing were performed at specialist reference laboratories as required.

Blood cultures were inoculated into BACT/ALERT culture media using an aseptic technique and incubated on the Biomerieux BACT/ALERT 3D Automated Microbial Detection system.

### Statistical Analysis

Frequencies of baseline characteristics including demographics, predisposing factors, admission GCS, and imaging were analyzed descriptively across the pediatric and adult cohorts. GCS on admission was converted into a categorical variable for analysis as follows: GCS 13–15 = good GCS; GCS 8–12 = moderate GCS; and GCS <8 = poor GCS. GOS was dichotomized into good outcome (GOS ≥4) or poor outcome (GOS ≤3). Microbiological data were divided into postsurgical and community-acquired cohorts for analysis. Frequencies of culture results across these 2 groups were analyzed descriptively.

Predictors of poor outcomes were defined a priori and included age, sex, GCS on admission, postsurgical infection, type of surgery, and infection with *S. anginosus*. A multivariate logistic regression model was used to examine for any association between these predictors and GOS and in-hospital mortality. Models were adjusted for age and sex.

Statistical analysis was performed using the STATA/MP 18.0 software package.

### Ethics

Data were anonymized before analysis and were collected and stored in accordance with relevant institutional and governance standards. Ethical approval was not required as all data were secured during standard clinical care.

## RESULTS

After excluding any erroneously coded records, there were 174 patients coded as having a primary admission involving a brain abscess. Two pediatric patients and 11 adult patients had >1 admission. Reasons for readmission included 1 adult patient who required re-operation 27 days after the date of initial surgery, 1 adult patient who was re-admitted 11 months after his primary admission with hydrocephalus, and 1 adult patient who developed a second brain abscess 4 years after his first presentation. Reasons for the other re-admissions were not available; however, all occurred within days to weeks of the original admission, and none involved surgery. Only the primary admission related to brain abscess has been included in the logistic regression analyses.

### Demographics

There were 32 pediatric patients aged <16 years (Table 1). Twenty-three were male (71.9%), and 9 were female (28.1%). The median age (interquartile range [IQR]) was 9.5 (0–13) years. Age was bimodally distributed, with 9 infants aged <1 year and 13 patients aged 10–15 years. There were 142 adult patients aged ≥16 years. Ninety-three were male (65.5%), and 49 were female (34.5%). The median age (IQR) was 53 (40–65) years. Across both the pediatric and adult cases, the ratio of male-to-female patients was 2:1.

**Table 1.** Baseline Characteristics of Pediatric and Adult Patients With Brain Abscess

	Pediatric Cohort, No. (%)	Adult Cohort, No. (%)	Total
Demographics			
No.	32 (18.4)	142 (81.6)	174
Age, median (IQR), y	9.5 (0–13)	53 (40–65)	N/A
Female	9 (28.1)	49 (34.5)	58 (33.3)
Male	23 (71.9)	93 (65.5)	116 (66.7)
Admission GCS			
Good	26 (81.3)	113 (79.6)	139 (79.9)
Moderate	4 (12.5)	14 (9.9)	18 (10.3)
Poor	2 (6.3)	6 (4.2)	8 (4.6)
Comorbidities			
Congenital heart disease	6 (18.8)	0 (0)	6 (3.4)
Hypertension	0 (0)	33 (23.2)	59 (0.3)
Malignancy	1 (3.1)	21 (14.8)	22 (12.6)
Solid organ transplant	0 (0)	3 (2.1)	3 (1.7)
Sinusitis	4 (12.5)	3 (2.1)	7 (4.0)
Valvular heart disease	0 (0)	9 (6.3)	9 (5.2)
Infective endocarditis	0 (0)	4 (2.8)	4 (2.3)
Dental infection	0 (0)	3 (2.1)	3 (1.7)
IDU	0 (0)	2 (1.4)	2 (1.1)
Diabetes mellitus	0 (0)	13 (9.2)	13 (7.5)
Alcohol excess	0 (0)	1 (0.7)	1 (0.6)
HIV	0 (0)	3 (2.1)	3 (1.7)
Imaging			
CT	5 (15.6)	29 (20.4)	34 (19.5)
MRI	8 (25.0)	19 (13.4)	27 (15.5)
CT & MRI	19 (59.4)	92 (64.8)	111 (63.8)
Abscess type			
Parenchymal	5 (15.6)	66 (46.5)	70 (40.2)
Subdural	16 (50.0)	24 (16.9)	37 (21.3)
Epidural	5 (15.6)	4 (2.8)	9 (5.2)
Postsurgical	1 (3.1)	31 (21.8)	36 (20.7)
Multiple	1 (3.1)	13 (9.2)	14 (8.0)
Other	3 (9.4)	2 (1.4)	5 (2.9)
>1 type	1 (3.1)	1 (0.7)	2 (1.1)

Abbreviations: CT, computed tomography; GCS, Glasgow Coma Score; IDU, injection drug use; IQR, interquartile range; MRI, magnetic resonance imaging.

### Predisposing Factors

Among the pediatric cohort, the most common predisposing factor was congenital heart disease (18.8%), followed by sinusitis (12.5%) and malignancy (3.1%) (Table 1). Malignancy (14.8%) and diabetes mellitus (9.2%) were the most common predisposing factors in the adult cohort. Nine adult patients had a diagnosis of valvular heart disease (6.3%). Infective endocarditis was uncommonly identified as a predisposing factor (2.8%). There were 3 adult patients with a recorded diagnosis of HIV infection (2.1%) and a further 3 who were solid organ transplant recipients (2.1%).

### Admission GCS

Most patients had a good GCS on admission (79.9%) (Table 1). GCS on admission was moderate in 10.3% and poor in 4.6% of cases. The pattern was similar across the pediatric and adult populations. Data on admission GCS were not available for 9 patients (5.2%).

### Imaging

Both CT and MRI scans were performed in most cases (63.8%) (Table 1). CT scan alone was performed in 19.5% of patients and MRI scan alone in 15.5%. No imaging records were available for 2 adult patients (1.1%). Rates of CT and MRI scanning were similar in both the pediatric and adult cohorts.

### Brain Abscess Type

In the pediatric cohort, subdural empyema was the most common abscess type (50.0%), followed by parenchymal and epidural abscesses (both 15.6%) (Table 1). In the adult cohort, parenchymal brain abscess was the most common abscess type (46.5%), followed by postsurgical brain abscess (21.8%) and subdural empyema (16.9%). Multiple abscesses were less frequent, occurring in 8.0% of the total cohort. Data on brain abscess type were not available for 9 patients (6.3%).

### Microbiological Data

There were 36 cases of postsurgical brain abscess (20.7%) and 138 cases of community-acquired brain abscess (79.3%) across the adult and pediatric cases (Table 2). A sample was received for diagnostic workup in the microbiology laboratory in 87.4% of cases. Gram-positive cocci were seen on 42.0% of gram stains, and no organisms were seen in 29.3%. A microbiological diagnosis was made in 129 cases across the total cohort (74.1%).

**Table 2.** Microbiology Results for Postsurgical and Community-Acquired Brain Abscess Cohorts. Individual Organisms Have Only Been Counted and Included When Identified as the Primary Pathogen in a Case. Only the Most Common Pathogens Are Presented in This Table

	Postsurgical, No. (%)	Community, No. (%)	Total, No. (%)
No. of cases	36 (20.7)	138 (79.3)	174
Microbiological diagnosis	28 (77.8)	101 (73.2)	129 (74.1)
Samples sent to microbiology			
Pus/tissue	29 (80.6)	102 (73.9)	131 (75.3)
CSF	2 (5.6)	5 (3.6)	7 (4.0)
Blood culture	1 (2.8)	8 (5.8)	9 (5.2)
Intraoperative swab	2 (5.6)	2 (1.4)	4 (2.3)
Ear swab	0 (0)	1 (0.7)	1 (0.6)
No sample received	2 (5.6)	20 (14.5)	22 (12.6)
Gram stain results			
No organism seen	18 (50.0)	33 (23.9)	51 (29.3)
Gram-positive cocci	9 (25.0)	64 (46.4)	73 (42.0)
Mixed	1 (2.8)	9 (6.5)	10 (5.7)
Gram-positive rods	1 (2.8)	1 (0.7)	2 (1.1)
Gram-negative rods	1 (2.8)	2 (1.4)	3 (1.7)
Fungal hyphae	0 (0)	1 (0.7)	1 (0.6)
Not performed/not available	5 (13.9)	28 (20.3)	33 (19.0)
Culture results			
*Staphylococcus aureus*	13 (36.1)	4 (2.9)	15 (8.6)
*Cutibacterium acnes*	5 (13.9)	0 (0)	5 (2.9)
Polymicrobial	3 (8.3)	12 (8.7)	15 (8.6)
*Streptococcus anginosus* group	0 (0)	62 (44.9)	62 (35.6)
16S RNA gene PCR results			
Samples sent	7 (19.4)	26 (18.8)	33 (19)
Significant pathogen identified	2 (5.6)	12 (8.7)	14 (8.0)

Abbreviations: CSF, cerebrospinal fluid; PCR, polymerase chain reaction.

**Figure 1.**
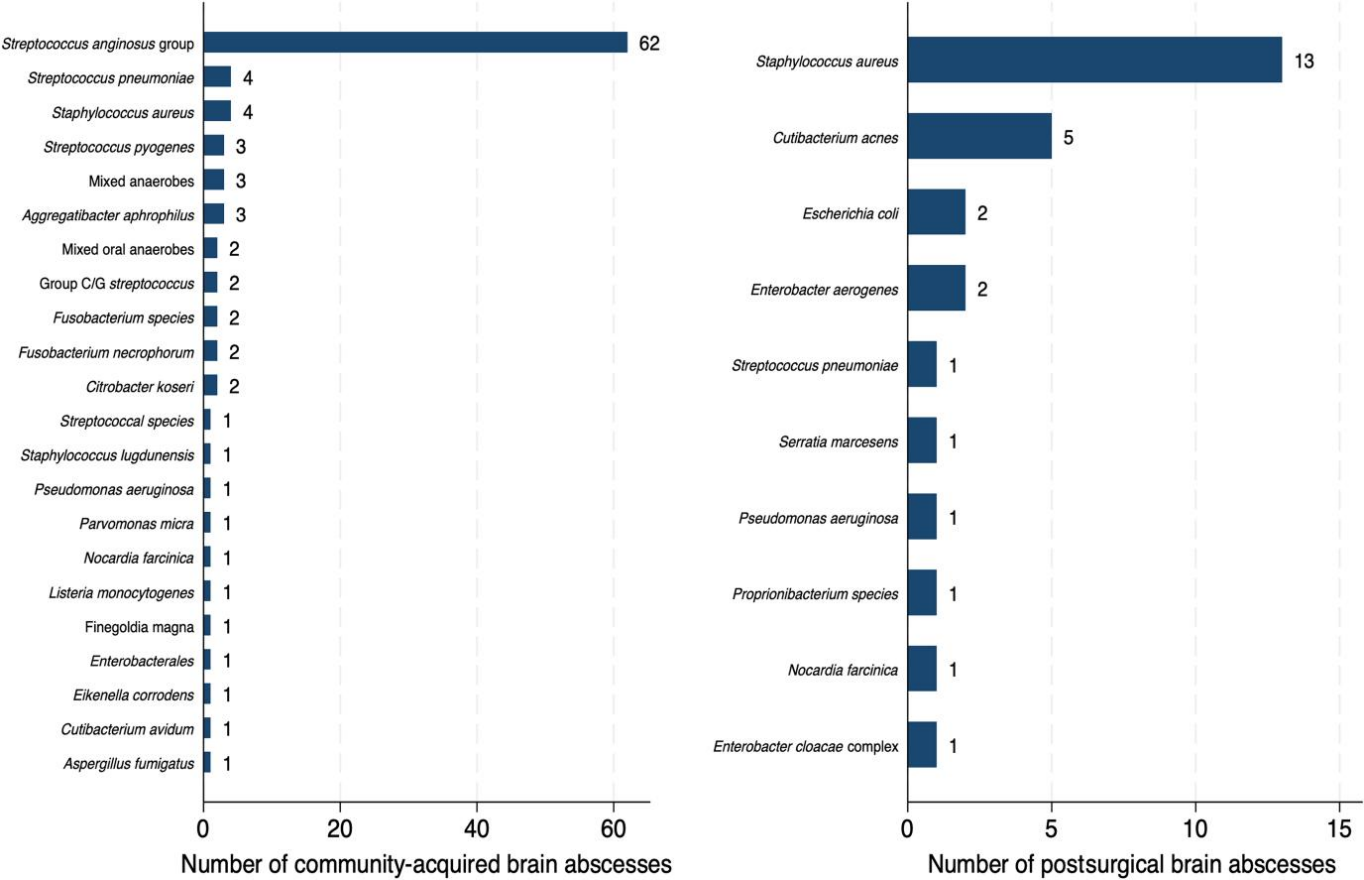
Frequency of causative pathogens of community-acquired and postsurgical brain abscess. For polymicrobial infections only, the primary pathogen is included.

**Figure 2.**
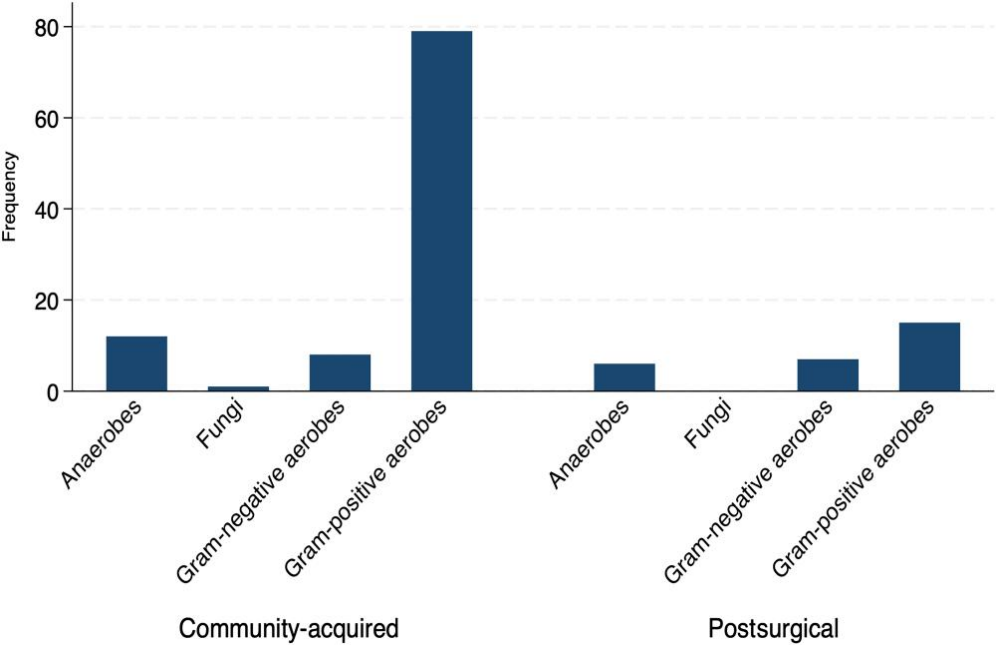
Frequency of causative pathogens of community-acquired and postsurgical brain abscess by microbiological grouping.

The most common causative pathogen in the postsurgical brain abscess cohort was *Staphylococcus aureus*, isolated in 13 of 36 cases in total (36.1%). Of these 13 cases, *S. aureus* was isolated as a sole pathogen in 10 cases (76.9%) and as part of a polymicrobial infection in 3 cases (23.1%). *Cutibacterium acnes* was the second most common pathogen, isolated in 5 (13.9%) cases as a sole pathogen only. Pathogens from the *Streptococcus anginosus* group were overwhelmingly the most common causative pathogens identified in the community-acquired brain abscess cohort, accounting for 62 (44.9%) cases; 58 of these cases involved the *S. anginosus* group as sole pathogens (93.5%). Polymicrobial infections accounted for a similar proportion of postsurgical and community-acquired cases and 8.6% of the total cohort (Supplementary Table 1). A more detailed breakdown of the primary causative pathogens identified in both groups can be seen in Figure 1, and the frequency of different pathogen groupings can be found in Figure 2.

The *S. anginosus* group was the most common bacterial cause of pediatric brain abscess in this study, accounting for 13 of 32 cases (40.6%). Of these 13 cases, 11 involved the *S. anginosus* group bacteria as sole pathogens (84.6%). *Streptococcus pyogenes* was isolated in 2 (6.3%) pediatric cases as a sole pathogen. *S*. *anginosus* group bacteria were also the most common cause of adult brain abscess, causing 50 out of 142 (34.5%) cases. Of these 50 cases, *S. anginosus* group bacteria were the sole pathogens in 48 (96.0%) cases. *S. aureus* was the second most common causative pathogen isolated in adults, causing 17 (12.0%) cases in total. Of these 17 cases, 14 (82.4%) involved *S. aureus* as a sole pathogen.

Samples were sent for 16S rRNA gene PCR in 33 of the 174 cases (19.0%). Twenty-five of these cases were ultimately culture-negative (75.8%). 16S rRNA gene PCR made a new microbiological diagnosis in 14 of these cases (42.4%), either by finding a single causative pathogen or by finding an additional pathogen that was not isolated with traditional microbiological culture. 16S rRNA gene PCR led to a modification of antimicrobial therapy in 5 of the 33 cases (15.2%). In each of these cases, the 16S rRNA gene PCR result would have enabled anaerobic cover with metronidazole to be stopped, reducing unnecessary antibiotic exposure. In 9 other cases, (27.3%), while the 16S rRNA gene PCR result would not have supported a change to the empirical management used in our institution, it would have confirmed that empirical treatment with ceftriaxone and metronidazole was correct. 16S rRNA gene PCR failed to identify 2 clinically significant organisms that grew on culture. In 2 other cases, it only identified 1 organism from a polymicrobial infection. Additional send-away PCR testing made a new microbiological diagnosis in 3 cases, 2 of *S. pneumoniae* and 1 of *Aspergillus fumigatus*.

One patient with a polymicrobial infection grew *Streptococcus constellatus* that was resistant to penicillin. All other streptococcal isolates were penicillin sensitive. There were no cases of methicillin-resistant *Staphylococcus aureus* (MRSA).

### Neurosurgical Data

**Figure 3.**
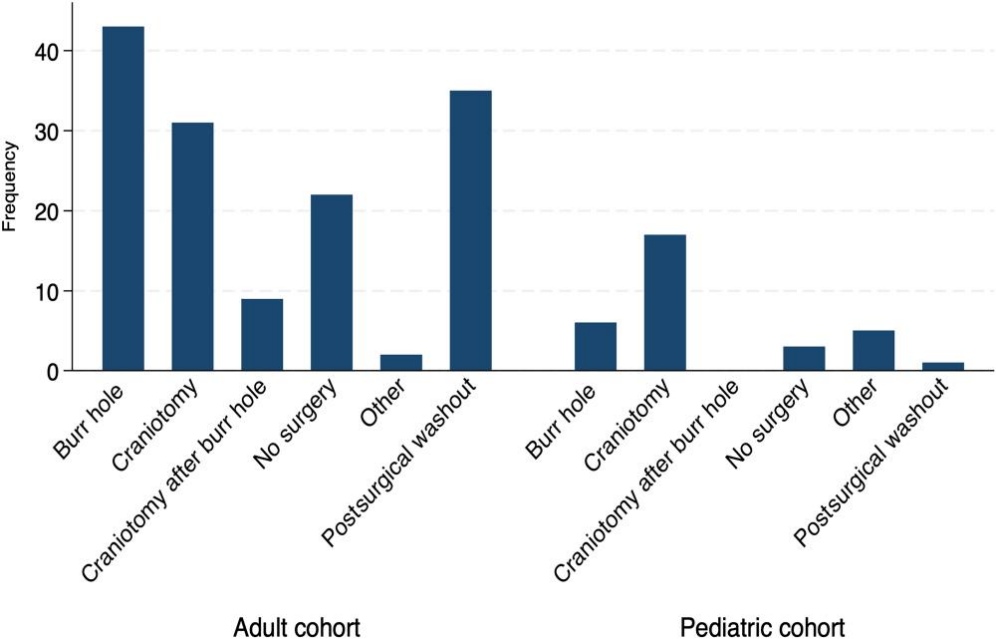
Frequency of surgical procedures in pediatric and adult cohorts.

Across the total cohort, 149 patients underwent surgery (85.6%) (Figure 3, Table 3). Craniotomy was the most common form of surgery in the pediatric cohort (53.1%), whereas burr hole was more common among adults (30.3%). Postsurgical washout was performed in 36 patients (20.7%). Among these 36 patients, the frontal and anterior skull base region was the most common site of original surgery, accounting for 12 (33.3%) postsurgical cases. Seven patients underwent other surgical procedures, for example, stent placement or ear, nose, and throat surgery only.

**Table 3.** Surgical Management and Outcomes for Pediatric and Adult Patients With Brain Abscess

	Pediatric Cohort, No. (%)	Adult Cohort, No. (%)	Total, No. (%)
Surgical procedure			
Craniotomy	17 (53.1)	31 (21.8)	48 (27.6)
Burr hole	6 (18.8)	43 (30.3)	49 (28.2)
Craniotomy after burr hole	0 (0)	9 (6.3)	9 (5.2)
Postsurgical washout	1 (3.1)	35 (24.6)	36 (20.7)
Other procedure only	5 (15.6)	2 (1.4)	7 (4.0)
No surgery	3 (9.4)	22 (15.5)	25 (14.4)
Mortality			
Died	0 (0)	19 (13.4)	19 (10.9)
Outcome (GOS)			
Good	31 (96.9)	109 (76.8)	140 (80.5)
Poor	1 (3.1)	25 (17.6)	26 (14.9)

Abbreviation: GOS, Glasgow Outcome Score.

### Outcome Data

In-hospital mortality among the adult cohort was 13.4%. There were no deaths in the pediatric cohort. Most patients had a good outcome (GOS ≥4; 80.5%). A good outcome was more common in the pediatric cohort than the adult cohort (96.9% and 76.8%, respectively). Outcome data were not available for 8 patients (4.6%).

### Statistical Analysis

Increasing age was a significant predictor of mortality, with each additional year of age increasing the odds of mortality by 6.0% in a sex-adjusted model (adjusted odds ratio [OR], 1.06; 95% CI, 1.03–1.09; *P* < .001). This association persisted when age was examined in the adult cohort only (OR, 1.06; 95% CI, 1.02–1.09; *P* = .002). Sex did not significantly associate with mortality in unadjusted or sex-adjusted models (adjusted OR, 1.02; 95% CI, 0.36–2.93; *P* = .969). Poor GCS on admission was associated with significantly higher mortality in both the unadjusted and age- and sex-adjusted models (adjusted OR, 7.73; 95% CI, 1.17–51.24; *P* = .034). Postsurgical brain abscess (adjusted OR, 1.20; 95% CI, 0.38–3.79; *P* = .762) and type of surgical procedure (craniotomy vs burr hole; adjusted OR, 0.52; 95% CI, 0.10–2.67; *P* = .432) were not significant predictors of mortality. Infection with *S. anginosus* was not significantly associated with mortality (adjusted OR, 0.28; 95% CI, 0.06–1.30; *P* = .104). Not undergoing surgery was associated with higher odds of mortality when adjusted for age and sex (adjusted OR, 3.03; 95% CI, 0.96–9.62; *P* = .060), though this result did not reach statistical significance.

## DISCUSSION

This is a large, retrospective series from a tertiary British neurosurgical center. It provides an overview of the epidemiology of postsurgical and community-acquired brain abscesses in children and adults over a 10-year period.

The ratio of male-to-female patients was 2:1, corroborating the preponderance of male patients with brain abscess described elsewhere in the literature [5, 13, 16–19], the reason for which remains unclear. Various explanatory factors have been proposed, including higher rates of trauma and relevant comorbidities in male patients [20] or a potential protective effect of female reproductive hormones [21]. The median age of adult patients was 53 years, in keeping with other case series [8, 13, 17–19, 22]. The ages of the pediatric cohort displayed a bimodal distribution, with most patients aged <1 year and between 10 and 15 years, a pattern noted in other studies [4]. This age distribution is likely to reflect predisposing factors to brain abscess that occur more commonly in these age groups, including uncorrected congenital heart disease in infants [23] and acute sinusitis in adolescents [24].

Congenital heart disease was noted in 18.8% of pediatric patients in this study, and sinusitis was reported in 12.5%. Malignancy was a predisposing risk factor in almost 15% of adult patients in this study. Previous research has shown that the adjusted odds ratios for brain abscess in people with solid organ and hematological malignancy are 4.1 and 8.8, respectively [8]. This is likely to reflect the immunosuppressive therapies used in the treatment of malignancy, the increased risk of hematogenous bacterial spread due to the use of intravenous catheters and disruption to the blood–brain barrier from chemotherapeutic agents, and the effects of the cancer itself. Brain abscess has also been associated with a substantially increased risk of cancer during the 10-year period following diagnosis [25], an observation that warrants further research.

A recorded diagnosis of dental disease was present in only 2.1% of adults in our cohort, despite dental disease being recognized as a major predisposing risk factor for brain abscess [8] and an estimated prevalence of dental caries among British adults of 31% [26]. The low rate of recorded dental disease likely reflects an underdiagnosis of dental disease in our institution. Members of the *S. anginosus* group are commensal bacteria of the human oral cavity and caused 42.8% of community-acquired brain abscess in this study, providing further evidence for an underdiagnosis of dental disease. This underdiagnosis may reflect declining rates of access to dental services in England, particularly since the coronavirus disease 2019 (COVID-19) pandemic [27], and a lack of confidence among doctors in diagnosing common oral conditions [28].

Increasing age was significantly associated with mortality in this study, in keeping with other published data [8]. We demonstrated a 6% increase in the odds of mortality with every additional year of age. This finding underscores the need for collaborative work between neurosurgical and medical teams, particularly those who specialize in elder care. Older patients are more likely to present with age-related physiological decline, multimorbidity, and cognitive impairment, increasing the risks of adverse surgical outcomes [29]. Interventions including comprehensive geriatric assessment have been shown to improve survival in patients undergoing elective surgery and should be evaluated in the acute neurosurgical setting [30]. Poor GCS on admission was another significant predictor of mortality independent of age, in keeping with other data that showed that GCS <14 was associated with poor neurological outcomes [31]. This likely reflects factors such as increased abscess size, poorer physiological function, and greater risk of irreversible neurological injury.

A microbiological diagnosis was made in 74.1% of cases. Reported rates of microbiological diagnosis in the literature vary between 33% and 92% depending on the center [19, 31]. Modern diagnostic methods used within our laboratory including MALDI-TOF and the advent of 16S rRNA gene PCR have likely contributed to the relatively high rates of pathogen identification seen in our study. Our results confirm the leading role played by the *S. anginosus* group of bacteria in community-acquired brain abscess, accounting for 43% of these cases. Similar patterns have been demonstrated in other recent case series [12, 14, 22, 31] both within the United Kingdom and internationally. The *S. anginosus* group consists of 3 distinct species, *S. anginosus*, *Streptococcus intermedius*, and *Streptococcus constellatus*. These bacteria are frequent colonizers of the human oropharynx and are known to cause dental, sinus, and ear nose and throat infections, underlining their role in the formation of brain abscesses. Their propensity toward abscess formation is likely a reflection of particular virulence factors including bacterial capsule, adhesins, and hydrolytic enzymes, although these remain incompletely understood [32]. The incidence of brain abscess due to *S. intermedius*, in particular, appears to be increasing. The reasons for this are unclear; however, improved diagnostics, an increase in virulent strains, increasing population carriage, rising source infections, demographic factors, and the COVID-19 pandemic may be contributing factors [33].

Previous research has demonstrated that brain abscesses due to *S. anginosus* group are significantly larger than those caused by other organisms [14]. Despite this, we observed reduced odds of mortality with brain abscesses caused by *S. anginosus* group, although the result did not reach statistical significance. This could be due to sample size limitations or may reflect the effects of unmeasured confounders. This observation warrants further investigation in larger studies to understand the potential differences in infection outcomes between different pathogens. We found only 1 case of penicillin-resistant streptococci in our study, supporting the recommendation of ceftriaxone plus metronidazole for first-line therapy in community-acquired brain abscess made in the recent ESCMID guidelines [10].

*S. aureus* was the most common causative pathogen of postsurgical brain abscesses in our study, while playing a minimal role in community-acquired infection. Over a quarter of postsurgical brain abscesses were caused by *S. aureus* compared with only 3.6% of community-acquired cases. These findings align with the well-established role of *S. aureus* as a nosocomial pathogen [7]. Reassuringly, we found no cases of MRSA in this study. This is in keeping with MRSA rates in our institution, where <5% of *S. aureus* isolates are methicillin-resistant. Postsurgical infection was not associated with mortality in this study. This may reflect earlier recognition and drainage in the postsurgical cohort as these patients are already under neurosurgical management.

*Cutibacterium acnes* emerged as a notable cause of postsurgical brain abscess in our cohort. *C. acnes* caused 13.9% of postsurgical cases, the second most common cause of postsurgical infection after *S. aureus*. *C. acnes* is a commensal of human skin and has been increasingly recognized as a cause of postneurosurgical infections [34] and brain abscess [35] despite its relatively indolent nature.

Despite the predominant role played by streptococci and *S. aureus* in this study, we also noted a broader range of pathogens including oral anaerobes and rarer pathogens including cases of *Nocardia farcinica*, *Listeria monocytogenes*, and *Aspergillus fumigatus*. The patient with brain abscess due to *L. monocytogenes* was 82 years of age and had monoclonal gammopathy of undetermined significance and diabetes mellitus. One patient with *Nocardia farcinica* brain abscess had a malignant brain tumor, and this was a postsurgical infection. The other patient with *N. farcinica* brain abscess was 79 years of age and had chronic obstructive pulmonary disease, interstitial lung disease, COVID-19 infection, and systemic lupus erythematosus. The patient with brain abscess due to *A. fumigatus* was a liver transplant recipient. Other studies have also noted an increasing proportion of rarer pathogens likely reflecting the improved availability and performance of microbiological diagnostic tests [22] as well as increasing use of immunosuppressive therapies.

In our study, 16S rRNA gene PCR made a new microbiological diagnosis in 14 cases, and other forms of PCR testing were diagnostic in 3 cases. Among the 33 cases where 16S rRNA gene PCR was performed, the result would have enabled the antibiotic regimen to be narrowed in 15.2% of cases. In 27.3% of cases, the result would have confirmed that empirical treatment with ceftriaxone and metronidazole was correct in an otherwise culture-negative case, potentially avoiding a switch to carbapenem and glycopeptide antibiotics, reducing broad-spectrum antibiotic exposure and facilitating antimicrobial stewardship. These results provide real-world evidence of the diagnostic utility of 16S rRNA gene PCR in brain abscess. Current guidelines conditionally recommend the use of molecular-based diagnostics in culture-negative cases [10]. Our work here supports the impact of molecular diagnostics on patient management, including choice and duration of antimicrobial therapy.

Almost 90% of patients in this study underwent some form of surgery, reflecting the importance of surgical source control in the management of brain abscess. Not undergoing surgery was associated with higher odds of mortality in this study, although this result did not reach statistical significance. Again, this may be due to sample size limitations. The observed association between undergoing surgery and improved in-hospital mortality may be confounded by selection bias, as patients who did not undergo surgery had significantly higher rates of frailty and multimorbidity. A trend toward improved survival was seen in patients who underwent a burr hole compared with craniotomy. Possible explanations may include a lower rate of postsurgical complications with less invasive surgery or selection bias, as people who undergo surgery via burr hole may have smaller abscesses.

Our study had several limitations. Our data rely on the accuracy of diagnostic coding, which may have resulted in some cases of genuine brain abscess at our center being missed. Due to the retrospective nature of our study, some data were missing. Our findings are applicable to a large, tertiary neurosurgical center in a high-income country, and caution must be applied before extrapolating them to other settings. Although antibiotic guidelines for brain abscess remained consistent over the study period, data on the specific antibiotic regimens received by individual patients were not available, and it was not possible to explore the effects of these on mortality. Finally, although our study had a larger sample size than other comparable cohorts, it may still have been underpowered to detect meaningful differences between smaller subgroups, for example, infection with *S. anginosus* or between different types of surgical procedures. Our study also has several strengths. We included both adult and pediatric patients and community-acquired and postsurgical brain abscesses, facilitating comparison across these groups. Data were collected and analyzed by both infection and neurosurgical specialists, allowing for cross-specialty expertise and discussion.

In conclusion, this large single-center study provides valuable insight into the contemporary epidemiology, microbiology, and outcomes of brain abscess in both pediatric and adult populations. *S. anginosus* group bacteria were the predominant cause of community-acquired brain abscess, while *S. aureus* was the most common pathogen in postsurgical cases. Increasing age and poor GCS at admission were key predictors of mortality, reinforcing the importance of early diagnosis and multidisciplinary management, particularly in older or more medically complex patients. Surgical intervention was associated with improved survival, reinforcing the importance of timely neurosurgical involvement. Molecular diagnostics, including 16S rRNA gene PCR, contributed to the microbiological diagnoses and may play an increasing role in guiding antibiotic therapy in the future. Further research is needed to explore optimal neurosurgical and antibiotic treatment strategies, particularly for patients deemed unsuitable for surgery. We support calls for a national registry of brain abscess cases in England to facilitate larger observational studies.

## Supplementary Material

ofaf655_Supplementary_Data

## Supplementary Data

Supplementary materials are available at *Open Forum Infectious Diseases* online. Consisting of data provided by the authors to benefit the reader, the posted materials are not copyedited and are the sole responsibility of the authors, so questions or comments should be addressed to the corresponding author.
