# Supplementary material for: Epidemiology of Brain Abscess: A Retrospective Cohort From a Neurosurgical Tertiary Referral Center in England
Source: Open Forum Infect Dis. 2025 Oct 21;12(11):ofaf655. doi: 10.1093/ofid/ofaf655 (PMC12625660; doi:10.1093/ofid/ofaf655)
Supplement: ofaf655_Supplementary_Data [file ofaf655_supplementary_data.docx]

| **Age** | **Sex** | **Post-Surgical Infection** | **Culture result - Isolate 1** | **Culture result - Isolate 2** | **Culture result - Isolate 3** | **Culture result - Isolate 4** | **Significant pathogen indentified on 16s / Other PCR** |  |
| --- | --- | --- | --- | --- | --- | --- | --- | --- |
| 22 | Male | Y | *Staphylococcus aureus* | *Citrobacter koseri* | N/A | N/A | N/A |  |
| 25 | Male | N | *Escherichia coli* | Mixed anaerobes | N/A | N/A | N/A |  |
| 42 | Male | Y | *Escherichia coli* | VRE | *Staphylococcus aureus* | N/A | N/A |  |
| 52 | Male | N | *Aggregatibacter aphrophilus* | *Parvomonas micra* | N/A | N/A | N/A |  |
| 50 | Male | N | *Aggregatibacter aphrophilus* | N/A | N/A | N/A | *Aggregatibacter aphrophilus + parvmonas micra* |  |
| 56 | Male | Y | *Staphylococcus aureus* | *Pseudomonas aeruginosa* | *Enterococcus faecalis* | N/A | N/A |  |
| 63 | Male | N | *Proteus mirabilis* | Anaerobes | *Enterococcus avium* | N/A | N/A |  |
| 64 | Female | N | *Fusobacterium necrophorum* | *Eggerthia catenaformis* | *Parvomonas micra* | *Actinomyces israelii* | N/A |  |
| 81 | Male | N | *Porphyromonas gingivalis* | *Tannerella forsythia* | N/A | N/A | N/A |  |
| 72 | Female | N | *Streptococcus anginosus group* | *Hameophilus parainfluenzae* | N/A | N/A | N/A |  |
| 69 | Male | N | *Streptococcus anginosus group* | *Staphylococcus aureus* | No | N/A | *Parvomonas micra* |  |
| 0 | Female | N | *Streptococcus anginosus group* | *Eikenella corrodens* | Anaerobes | N/A | N/A |  |
| 11 | Male | N | *Streptococcus anginosus group* | *Bacteroides fragilis* | N/A | N/A | N/A |  |
| 13 | Male | N | *Staphylococcus lugdunensis* | Staphyloccus epidermidis | N/A | N/A | N/A |  |
| 0 | Female | N | *Staphylococcus aureus* | N/A | N/A | N/A | *Streptococcus pneumoniae* |  |

*Supplementary Table 1. Detailed results of polymicrobial brain abscess cases*
